# Evaluating the efficiency of isoflurane capture from anaesthetised experimental sheep: a proof‐of‐concept prospective observational study for a means of reducing emissions in research animals

**DOI:** 10.1111/anae.16702

**Published:** 2025-07-21

**Authors:** Florence E. Hillen, Jane S. McLaren, Kate L. White

**Affiliations:** ^1^ University of Nottingham Nottingham UK

The controlled conditions of experimental animal anaesthesia provide an ideal opportunity to develop and refine carbon‐saving practices. Emissions of inhalational anaesthetic agents account for approximately 3% of the healthcare‐associated footprint [1]. The environmental impact of animal research is unknown, but inhalation anaesthesia is commonplace. Carbon‐based capture devices have the potential to reduce atmospheric release with the aim of creating a circular economy by desorbing the compounds for reuse. In vitro mass transfer rates of inhalational anaesthetic agents for marketed devices are reportedly 99–100%, but under clinical conditions, capture efficiencies of 25–51% have been obtained from human subjects [2, 3, 4] suggesting that patient factors, practices, and equipment are responsible for the inefficiencies of the processes. Proof‐of‐concept for anaesthetic agent capture in an experimental animal could lead to future reductions in emissions from the animal research industry and, in a species of similar body size to an average adult human, aid in identifying factors affecting in vivo mass transfer and the development of improved medical practices. Appropriate translation could, therefore, lead to significant carbon savings within the healthcare sector.

The University of Nottingham Animal Welfare and Ethical Review Body granted ethical approval to capture isoflurane from experimental sheep undergoing general anaesthesia for bone defect creation using an activated carbon device, VET‐can (SageTech, Paignton, UK). The vapourisers and VET‐can were weighed using a precision balance (CGOLDEN‐WALL, Feng Zhen, China) before and after each procedure. In vivo mass transfer proportion (weight gain of the VET‐can divided by the sum of the weight reduction of the vaporisers used) and capture efficiency (mass of isoflurane desorbed as a proportion of the mass of isoflurane used) were computed. The VET‐can was positioned within the scavenging system. The exhaust of the anaesthetic monitor was also connected to the inlet device and a sample of anaesthetics was selected until the device was filled to > 99% capacity (required for efficient desorption). Following induction of anaesthesia (online Supporting Information Appendix S1), the tracheas were intubated with a cuffed silicone tracheal tube. Anaesthesia was maintained with isoflurane in 100% oxygen (3–4 l.min^‐1^) via a circle breathing system. Lungs were ventilated to normocapnia (Matrix 3000, Midmark Animal Health USA, Kansas City, KS, USA). Spearman's ρ correlation analysis of anaesthetic‐ and animal‐related variables and in vivo mass transfer was performed using SPSS (version 29.0.2.0 [20], IBM, Armonk, NY, USA). Carbon saving from preventing atmospheric release of isoflurane was estimated (specific gravity at 20°C: 1.5 g.ml^‐1^, carbon conversion of isoflurane: 0.806 kg CO_2_e.ml^‐1^ [5]).

Isoflurane was captured from 16 anaesthetics (Table 1). Capture took place in the operating theatre and the preparation room for 12 anaesthetics and in theatre for four anaesthetics due to a shortage of personnel available to move and reconnect the equipment. The proportion capture time of overall inhalational anaesthesia time was calculated and included in the univariate exploratory analysis. VET‐can filling was completed part way through the final anaesthetic which was excluded from the investigation of anaesthetic‐ and animal‐related variables. An in vivo mass transfer of 27% was obtained from this animal.

**Table 1:** Patient variables, anaesthetic and capture data and correlation with in vivo mass transfer for the 15 sheep included in the univariate exploratory analysis. Values are median (IQR [range]).

In vivo mass transfer (%)			
All animals			70 (22 [38–90])
Isoflurane captured in operating theatre only (n = 4)			46 (13.5 [38–55])
Isoflurane captured in preparation room and operating theatre (n = 11)			75 (12 [60–90])

Correlation between anaesthetic‐ and animal‐related factors and in vivo mass transfer			
		Spearman's ρ correlation	p value
Weight; kg	63 (5 [57–79])	‐0.29	0.300
Duration of anaesthesia; min	105 (15 [95–135])	0.01	0.960
Capture of total inhalation anaesthesia time; %*	100 (26 [70–100])	0.71	0.003^¶^
End‐tidal isoflurane; %	1.1 (0.10 [0.9–1.4])	‐0.17	0.550
MAC multiples**	0.75 (0.06 [0.58–0.91])	‐0.06	0.840
MAC hours^†^	1.30 (0.34 [1.02–2.06])	‐0.09	0.750
Hypotensive index (60)^‡^	0 (1.67 [0–5.25])	‐0.12	0.680
Hypotensive index (70)^§^	1.2 (5.83 [0–17.5])	0.09	0.760
Mean arterial blood pressure; mmHg	88 (8 [68–111])	‐0.12	0.670
End‐tidal carbon dioxide; kPa	6.4 (1.33 [5.3–7.7])	‐0.15	0.600
SPO_2_; %	100 (1.0 [94–100])	‐0.43	0.110

* Duration of isoflurane capture/total time over which general anaesthesia was maintained with isoflurane inhalation.

** End‐tidal isoflurane/MAC isoflurane (1.5%, [6]).

^†^ [(Mean end‐tidal isoflurane concentration/MAC isoflurane (1.5%)) × hours of isoflurane anaesthesia].

^‡^ [(60 mmHg ‐ mean arterial pressure) × time in hours that mean arterial pressure < 60 mmHg].

^§^ [(70 mmHg ‐ mean arterial pressure) × time in hours that mean arterial pressure < 70 mmHg].

For Spearman's ρ correlation interpretation of association: 0.00–0.19, very weak; 0.20–0.39, weak; 0.40–0.59, moderate; 0.60–0.79, strong; 0.80–1.00, very strong. Results were considered significant where p = < 0.05

The mass of isoflurane used was 875.9 g and the weight gain of the VET‐Can was 557.9 g. In total, 537 g of liquid was extracted (492 g isoflurane, 92%; 45 g water, 8%) representing 107% fill. Capture efficiency across the 16 anaesthetics was 56% with a median in vivo mass transfer of 70%. Carbon saving was 3.08 kg CO_2_e.20 min^‐1^ of anaesthesia time. When backwards translation was used to remove the last sheep, assuming a constant isoflurane to water ratio across anaesthetics, efficiency increased to 58%. By the same reasoning, only including anaesthetics where isoflurane was captured in both the operating theatre and preparation room suggested capture efficiency would be 63%.

Compared with existing reports, we showed a capture efficiency higher than in humans [2, 3] and lower than in cats and dogs [7]. Unsurprisingly, there was a strong correlation between the proportion of inhalational anaesthesia time over which capture occurred, suggesting that ensuring capture throughout the anaesthetic period and limiting periods of disconnection could improve efficiency. Association between in vivo mass transfer and hypotension has been reported previously [7], but we did not observe this. In contrast with the aforementioned report, incidences of hypotension occurred early in the anaesthetic period, were treated aggressively and resolved quickly. Optimising blood pressure management may be another strategy to improve capture efficiencies, presumably by improving tissue perfusion and increasing the washout rate. Rapid adoption of low fresh gas flows [8] and comparatively lower end‐tidal volatile agent at tracheal tube disconnection [4] could have improved capture efficiencies, but were not assessed in our study.

The small sample size, similarities in patient characteristics and single anaesthetic protocol tested are limitations. However, the findings provide proof‐of‐concept for inhalational anaesthetic agent capture in sheep. This species is used in orthopaedic, cardiovascular, respiratory, gastrointestinal and neurodegenerative disease research, suggesting that a range of clinical scenarios could be mimicked, thus facilitating future evaluation and optimisation of inhalational agent capture for translation to human medicine.

## Supporting information


**Appendix S1.** Anaesthesia protocol.
