# Supplementary material for: Evaluating the efficiency of isoflurane capture from anaesthetised experimental sheep: a proof‐of‐concept prospective observational study for a means of reducing emissions in research animals
Source: Anaesthesia. 2025 Jul 21;80(9):1146–8. doi: 10.1111/anae.16702 (PMC12351216; doi:10.1111/anae.16702)
Supplement: Supplementary file 1 — Appendix S1. Anaesthesia protocol. [file ANAE-80-1146-s001.docx]

**Appendix S1:** Anaesthesia protocol

**Animals**

Sixteen healthy (ASA I) cross-bred (mule) female entire sheep, aged 2-4 years.

General anaesthesia for experimental bone defect creation of femoral condyles.

February 2025, University of Nottingham Biosciences Unit

**Pre-anaesthetic management**

Group housed, moved to holding pen with a companion the day before surgery, food was withdrawn 12 hours prior to anaesthesia and water at the time of premedication.

**Premedication**

Methadone 0.2 mg.kg^-1^ (Comfortan, Dechra, UK) IM

Xylazine 25-50 mg.kg^-1^ (Rompun 2%, Dechra, UK) IM

Dorsal surface of pinnae clipped and lidocaine/ prilocaine (EMLA 5% cream, Aspen, UK) applied.

**Induction of anaesthesia**

Ketamine 1.5-5.0 mg.kg^-1^ (Anesketin, Dechra, UK) IV

Midazolam 0.2-0.35 mg kg^-1^ (Dormazolam, Dechra, UK) IV

**Tracheal intubation**

Larynx desensitised with 1 ml lidocaine hydrochloride 2% w/v (Lidocaine hydrochloride injection, Hameln, UK).

Trachea intubated with 10-12mm silicone tracheal tube.

Tracheal cuff inflated to 30 cmH_2_O (AG Cuffill, Hospitch Respiration, Israel).

**Maintenance of anaesthesia**

Isoflurane carried in 100% oxygen via circle breathing system, ET_ISO_ 1.1-1.3%

Allowed to breathe spontaneously in the prep room with manual intermittent positive pressure ventilation as required to maintain ETCO_2_ < 8 kPa. Controlled mechanical ventilation to maintain normocapnia (ETCO_2_ 4.7-6kPa) in theatre (Matrix Model 3000, Midmark Animal Health, USA).

Meloxicam 1 mg.kg^-1^ IV following induction of anaesthesia.

Constant rate infusion of ketamine 5 mg.kg^-1^.min^-1^ IV.

Ketamine 0.5-1.0 mg.kg^-1^ and fentanyl 0.5-1.0 µg.kg^-1^ (Fentadon, Dechra, UK) administered to improve the plane of anaesthesia/ treat nociception as appropriate.

Dobutamine, atropine and adrenaline available for management of hypotension.

**Local anaesthesia**

Bilateral incisional local anaesthetic blocks with 5 ml lidocaine 2% w/v per side.

Or unilateral incisional block with lidocaine 2% w/v and a unilateral greater ischiatic notch plane combined with a caudal quadratus lumborum (GIN-TONIC) block with 5 ml 1% w/v lidocaine at each injection site (animals simultaneously enrolled on a separate pilot study assessing the feasibility and efficacy of this block in sheep).

**Intravenous fluid therapy**

Lactated Ringer’s solution (Vetivex 11 (Hartmann’s) Solution, Dechra, UK) 5ml.kg^-1^ hr IV.

Increased for management of hypotension.

**Instrumentation and monitoring**

20-G venous cannula into a lateral auricular vein for venous access.

6Fr, 20 cm central venous catheter (Infusion Concepts, Halifax, UK) into left or right jugular vein for venous access and blood sampling.

20-gauge arterial cannula into a radial or auricular artery for direct arterial blood pressure measurement.

Blood pressure cuff over metatarsal artery for oscillometric non-invasive blood pressure monitoring (Datex-Ohmeda S/5, GE Healthcare, Finland).

Side-stream capnography

Three-lead ECG for evaluation of the echocardiogram.

Pulse oximeter probe applied to the tongue.

Oesophageal temperature probe.

Venous and arterial blood gas analysis were available if required (EPOC, Siemens Healthineers, Germany)

[Animals were monitored continuously, and data were recorded every 5 minutes]

**Antibiotics**

Penicillin 8 mg.kg ^-1^ and streptomycin 10 mg.kg^-1^ (Pen & Strep, Norbrook, UK) IM administered 30 min prior to incision and repeated once daily for 2 days postoperatively.

**Recovery**

Sheep were recovered in sternal recumbency.

The tracheal tube and pharynx were suctioned to remove any saliva and any regurgitated ruminal contents to prevent aspiration.

The trachea was extubated in theatre once sheep were swallowing or chewing.

Animals were moved to a recovery pen, encouraged to eructate and offered a small amount of concentrate and *ad lib* hay.

Sheep remained in the recovery pen overnight with a companion.

The neck was bandaged to prevent damage to or contamination of the central venous catheter.

**Postoperative analgesia**

Pain scores (Sheep Grimace Scale^1^ and locomotion scoring scale^2^) were performed twice daily post-operatively and an additional dose of meloxicam 1 mg.kg^-1^ i.v was administered 48 hours post-surgery.

**References**

1. Häger C, Biernot S, Buettner M, et al. The Sheep Grimace Scale as an indicator of post-operative distress and pain in laboratory sheep. *PLoS One* 2017; **12**: 1-15. https://doi: 10.1371/journal.pone.0175839.
2. Kaler J, Wassink GJ, Green LE. The inter- and intra-observer reliability of a locomotion scoring scale for sheep. *Vet J* 2009; **180**: 189-194. https://doi: 10.1016/j.tvjl.2007.12.028.
